# p15^PAF^ Is an Rb/E2F-Regulated S-Phase Protein Essential for DNA Synthesis and Cell Cycle Progression

**DOI:** 10.1371/journal.pone.0061196

**Published:** 2013-04-04

**Authors:** Chih-Ning Chang, Mow-Jung Feng, Yu-Ling Chen, Ray-Hwang Yuan, Yung-Ming Jeng

**Affiliations:** 1 Graduate Institute of Pathology, National Taiwan University, Taipei, Taiwan; 2 Department of Surgery, National Taiwan University Hospital, Taipei, Taiwan; 3 Department of Pathology, National Taiwan University Hospital, Taipei, Taiwan; Virginia Tech, United States of America

## Abstract

The p15^PAF^/KIAA0101 protein is a proliferating cell nuclear antigen (PCNA)-associated protein overexpressed in multiple types of cancer. Attenuation of p15^PAF^ expression leads to modifications in the DNA repair process, rendering cells more sensitive to ultraviolet-induced cell death. In this study, we identified that p15^PAF^ expression peaks during the S phase of the cell cycle. We observed that p15^PAF^ knockdown markedly inhibited cell proliferation, S-phase progression, and DNA synthesis. Depletion of p15^PAF^ resulted in p21 upregulation, especially chromatin-bound p21. We further identified that the p15^PAF^ promoter contains 3 E2F-binding motifs. Loss of Rb-mediated transcriptional repression resulted in upregulated p15^PAF^ expression. Binding of E2F4 and E2F6 to the p15^PAF^ promoter caused transcriptional repression. Overall, these results indicate that p15^PAF^ is tightly regulated by the Rb/E2F complex. Loss of Rb/E2F-mediated repression during the G1/S transition phase leads to p15^PAF^ upregulation, which facilitates DNA synthesis and S-phase progression.

## Introduction

The p15^PAF^ protein, also known as KIAA0101 or OEATC-1, is a 15-kDa nuclear protein initially identified using a yeast two hybrid screen for proteins that bind to proliferating cell nuclear antigen (PCNA) [1]. p15^PAF^ binds to PCNA through the conserved PCNA-interacting protein motif (PIP box, Qxx(L/I/M]xx[F/Y][F/Y]) at amino acids 62–69 (62-QKGIGEFF-69) [1]. No other functional domains or motifs have been identified using bioinformatic methods. p15^PAF^ is overexpressed in multiple types of human cancer, including hepatocellular carcinoma [2], lung cancer [3], breast cancer [4], and pancreatic cancer [5], and its overexpression is associated with poor patient outcome [2]–[4]. Overexpression of p15^PAF^ promotes cancer cell growth, whereas attenuation of expression by small interfering RNA (siRNA) leads to reduced cell proliferation [5]. These results indicate that p15^PAF^ has a growth-promoting role; however, the molecular mechanisms underlying its effects have yet to be identified.

p15^PAF^ was reported to play a role in DNA repair. In a study by Simpson et al., p15^PAF^ expression was upregulated in response to ultraviolet (UV) irradiation. Besides, the associations of p15^PAF^ and p33^ING1b^ with PCNA were enhanced after UV irradiation [6]. Similar to p15^PAF^, p33^ING1b^ is a PCNA-interacting protein, and is an isoform encoded by the ING1 tumor suppressor locus. Overexpression of p33^ING1b^ confers increased efficiency of repair of UV-damaged DNA in melanoma cells, and p53 is required for the repair process [7]. Overexpression of p15^PAF^ also protects cells from UV-induced cell death [6]. p15^PAF^ is a direct transcriptional target of ATF3. ATF3 and p15^PAF^ expression are sufficient to trigger the DNA repair machinery against UV damage [8]. p15^PAF^ was also reported to interact with BRCA1 and regulate the centrosome number [9]; however, it is not yet known if p15^PAF^ is a component of the double-strand break repair pathway.

Several PCNA-interacting proteins are regulators of cell cycle progression or components of DNA synthesis machinery [10]. In this study, we evaluated the role of p15^PAF^ in cell cycle progression and DNA synthesis, and showed that p15^PAF^ is a direct transcriptional target of the Rb/E2F pathway.

## Materials and Methods

### Immunohistochemical stain

The tissue distribution of p15^PAF^ protein was evaluated using a tissue array containing adult tissues of major organs, obtained from normal regions of surgically resected patient specimens. A 2 mm tissue core was removed for each organ using a manual tissue array device (Beecher Instruments, Silver Spring, MD, USA) and inserted into a recipient paraffin block. The arrayed tissues were cut into 4 µm slices and placed on positively charged slides. Tissue sections were dewaxed and rehydrated. Antigen retrieval was performed by incubating slides in 0.01 M citric acid buffer (pH 6.0) at 100 °C for 10 min. After blocking with 3% hydrogen peroxide and 5% fetal bovine serum (FBS), slides were incubated with a monoclonal antibody against p15^PAF^ (Abnova, Taipei, Taiwan) at 1∶500 dilution at 4 °C overnight. Slides were then incubated with polymer-horseradish peroxidase (HRP) reagent (BioGenex, San Ramon, CA, USA). Peroxidase activity was visualized using diaminobenzidine tetrahydroxychloride solution (BioGenex). Sections were counterstained with hematoxylin. Dark brown nuclear staining was defined as positive, and no staining was defined as negative. For negative controls, the primary antibody was replaced with 5% FBS. This study was approved by the Research Ethical Committee of National Taiwan University Hospital. Written informed consent was obtained from all patients.

### Cell culture and treatment

HeLa cells, MCF7 cells, and viral package 293T cells were cultured in Dulbecco's modified Eagle's medium supplemented with 10% FBS in an incubator at 37 °C, with a humidified atmosphere of 95% air and 5% carbon dioxide (CO_2_). For synchronization of the cell cycle, HeLa cells were treated with 2 mM thymidine (Sigma-Aldrich, St. Louis, MO, USA) for 14 h and then washed 3 times with phosphate buffered saline (PBS). They were then incubated in normal growth medium for 10 h prior to treatment with aphidicolin (5 µg/mL; Sigma-Aldrich) or thymidine (2 mM) for an additional 14 h to arrest cells at the G1/S boundary.

### Cell proliferation assay

The 3-(4,5-dimethylthiazol-2-yl)-2,5-diphenyl-2H-tetrazoliumbromide (MTT) assay was used to evaluate the rate of cell proliferation. This colorimetric assay measures the activity of cellular enzymes that reduce MTT dye to insoluble MTT formazan, giving a purple color. One-thousand living cells were seeded into 96-well plates and incubated at 37 °C in a humidified atmosphere with 5% CO_2_. After an appropriate time interval, MTT was added and incubated for 4 h. The resulting color reaction product was extracted using dimethyl sulfoxide and absorbance was measured at 570 nm.

### RNA interference

For the knockdown of endogenous p15^PAF^, the target sequences p15-4: 5′- GCAACCTGATCACACAAATGA -3′ and p15-5: 5′- GCTTTGTTGAACAGGCATTTA -3′ were constructed in the shRNA vector pLKO.1. An shRNA vector against luciferase (pLKO.1-shLuc) was used as a negative control. For lentivirus production, 293T cells were transfected with 4 µg pLKO.1 lentiviral vector, along with 0.4 µg envelope plasmid pMD.G and 3.6 µg packaging plasmid pCMVΔR8.91. Viruses were collected 24 h and 48 h post-transfection. To prepare p15^PAF^ knockdown cells, HeLa cells were infected with the lentivirus for 24 h. Fresh medium containing 2 µg/mL puromycin (Sigma-Aldrich) was added for 2 d for drug-resistant cell selection.

Rb was knocked down by transfection using siRNA duplexes purchased from Life Technologies (Carlsbad, CA, USA): siRb-1, 5′-ACAGAAGAACCUGAUUUUATT-3′ and siRb-2, 5′-GAUACCAGAUCAUGUCAGATT-3′.

### RNA isolation and reverse transcription-polymerase chain reaction (RT-PCR)

Total RNA was extracted with Trizol reagent (Life Technologies). RT-PCR was used to determine p15^PAF^ and p21 mRNA expression using ribosomal protein S26 mRNA as the internal control. PCR was arrested during the exponential phase, 26 cycles for p15^PAF^, 28 cycles for p21, and 22 for S26. PCR was performed in an automatic DNA thermal cycler, with initial heating at 94 °C for 2 min, followed by cycles of 94 °C for 30 s, 60 °C for 1 min, 72 °C for 1 min, and final 72 °C extension for 10 min. The primers for p15^PAF^ were p15-F: 5′-AAAGCATGTCCTTTGCAACC-3′ and p15-R: 5′-TGGCACCATTCCAATAATCA-3′. The primers for p21 were p21-F: 5′-GCGCCATGTCAGAACCGGCTGG-3′ and p21-R: 5′-TTAGGGCTTCCTCTTGGAGA -3′.The primers for S26 were S26-F: 5′-CCGTGCCTCCAAGATGACCAAAG-3′ and S26-R: 5′-GTTCGGTCCTTGCGGGCTTCAC-3′.

### Flow cytometry

The harvested cells were resuspended in 100 µL PBS at a density of 2×10^6^ cells/mL. The cells were added dropwise to 1 mL ice-cold 70% alcohol for fixation at −20°C overnight. The cells were then washed twice with PBS and incubated with 1 mL of PBS containing 10 mg/mL RNase A and 1 mg/mL propidium iodide (Sigma-Aldrich) for 30 min to degrade RNA and stain DNA. DNA ploidy was analyzed using a FACSCalibur flow cytometer (BD Biosciences, San Jose, CA, USA).

### BrdU incorporation assay

Incorporation of BrdU during the S phase was evaluated using the BD BrdU FITC Assay Kit (BD Biosciences) according to the manufacturer's instructions.

### Immunofluorescence

The HeLa cells were seeded onto coverslips at 60%–80% confluency and incubated overnight. After washing twice with PBS, cells were fixed with 4% paraformaldehyde in PBS for 10 min, permeabilized with 0.1% PBS with Tween 20 (PBST) for 10 min, and then blocked in PBS containing 0.5% bovine serum albumin (BSA) for 30 min, and incubated with the anti-p15^PAF^ antibody (1∶5000, Abnova) at 4 °C overnight. Cells were washed 3 times with 0.1% PBS and then incubated with goat anti-mouse IgG or goat anti-rabbit IgG secondary antibody coupled to Alexa® 488 or Alexa® 594 dyes (1∶1000, Life Technologies) for 1 h. Nuclei were stained using 4′,6′ diamino-2-phenylindole (DAPI) (1 µg/µL). Confocal imaging was performed using a Zeiss LSM 510 META laser-scanning microscope.

For detection of incorporated BrdU, cells were pulsed with BrdU (10 µM, Roche, Basel, Switzerland) for 30 min at 37 °C and harvested at the indicated time points prior to fixation. Cells were fixed with cold methanol for 20 min at −20°C. DNA was denatured by treating with 4 M hydrogen chloride for 15 min and washed 3 times with PBS. Immunofluorescence was performed as described using FITC-anti-BrdU antibody (1∶100, AbCam, Cambridge, MA, USA).

### Western blotting

Protein samples (50 µg each) were separated using 10%–15% SDS-PAGE and then electrotransferred onto nitrocellulose membranes (Amersham, Buckinghamshire, UK). The membranes were allowed to react with the primary and secondary antibodies at optimum dilution, and the immunoreactive signals were detected using an enhanced chemiluminescence kit (Millipore, Bedford, MA, USA). The antibodies used included p15^PAF^ (1∶1000, Abnova), p21 (1∶1000, Santa Cruz Biotechnology, Santa Cruz, CA, USA), β-actin (1∶5000, Sigma-Aldrich), Rb (1∶1000, 11D7, generously provided by Dr. Wen-Hwa Lee, University of California, Irvine, CA), cyclin D1 (1∶1000, Neomarkers, Fremont, CA, USA), cyclin B1 (1∶1000, Neomarkers), and PCNA (1∶1000, Neomarkers).

### Reporter assay

The *p15^PAF^* promoter fragment −208 to +94 bp was obtained using PCR. The primers used were p15^PAF^/SacI-208: 5′-AGAGCTCATTTCTGTAGTTCAGAAAC-3 and p15^PAF^/HindIII+94: TAAGCTTCCCAGCCGAGGGTGTTTC. The PCR fragments were cloned into a promoterless pGL3-basic vector. Mutations were introduced by site-directed mutagenesis in the putative E2F-binding sites using the QuickChange site-directed mutagenesis kit (Stratagene, La Jolla, CA, USA). The *AURKA* and *MCL1* promoters, E2F4 and E2F6 expression plasmids, were gifts from Dr. Ju-Ming Wang (Institute of Bioinformatics and Biosignal Transduction, National Cheng Kung University). The Rb expression vector pSLX-CMV-Rb was kindly provided by Dr. Ming-Fu Chang (Institute of Biochemistry and Molecular Biology, National Taiwan University). Transient transfection was performed using the Turbofect reagent (Thermo Fisher, Waltham, MA, USA). Reporters were cotransfected with expression vectors of Rb, E2F4, E2F6 or the control plasmid, and the *Renilla* luciferase plasmid TK-Renilla, into MCF7 cells, which were grown in 12-well plates at 70% confluence. Twenty-four h after transfection, cell extracts were prepared, and luciferase activities were quantified using the Dual-Glo Luciferase Assay System (Promega, Madison, WI, USA) in an Orion II luminometer (Berthold Detection Systems, Pforzheim, Germany). All experiments were performed in triplicate.

### Chromatin immunoprecipitation

After culturing to 60%–70% confluency, formaldehyde (1% final concentration) was added for 10 min crosslinking, and then glycine (125 mM final concentration) was added to arrest crosslinking. DNA was sheared by sonication. The sheared chromatin fragments were immunoprecipitated with an antibody specific for E2F4 (Thermo Scientific), E2F6 (Abcam), or control IgG (Santa Cruz) at 4 °C for 16 h. The antibody-chromatin complexes were precipitated using protein-G-sepharose slurry (Sigma-Aldrich) at 4 °C for 1 h. After dissociation from the immunoprecipitated chromatin, DNA underwent PCR amplification using primers for GAPDH and the putative E2F-binding sites.

### Chromatin-extracted immunofluorescence

Control and p15^PAF^ knockdown HeLa cells were seeded onto coverslips at 60%–70% confluency and incubated overnight. After washing twice with PBS, cells were extracted with CSK buffer (10 mM PIPES-KOH (pH 7), 100 mM NaCl, 300 mM sucrose, 3 mM MgCl_2_, and 0.5% Triton X-100) and fixed with 4% paraformaldehyde. After blocking with PBS containing 0.5% BSA for 30 min, the cells were incubated with the indicated primary antibodies at 4 °C overnight. They were then washed 3 times with 0.5% PBST and incubated with goat anti-mouse IgG or goat anti-rabbit IgG secondary antibody coupled to Alexa® 488 or Alexa® 594 dyes (1∶1000, Life Technologies) for 1 h. Nuclei were stained using DAPI (1 µg/µL). Confocal imaging was performed using a Zeiss LSM 510 META laser-scanning microscope. The primary antibodies used were p15^PAF^ (1∶5000, Abnova) and p21 (1∶500, Santa Cruz).

### GST-tagged protein-protein interaction assay

Full-length open-reading frames of p15^PAF^ and p21 were cloned to pET-15b (Novogen, Madison, WI, USA). His-tagged proteins were expressed in *E.Coli* strain BL21 (DE3) (Stratagene) and purified using nickel ion chromatography. pGEX-4T-1 containing the full-length open-reading frame of PCNA was transformed into BL21, and PCNA-GST fusion protein expression was induced using 0.5 µM isopropyl-D-1-thiogalactopyranoside (IPTG) for 4 h at 18 °C. The GST fusion proteins were purified using glutathione-sepharose beads (GE Healthcare, Little Chalfont, UK). His-tagged p21 (25 ng) and different amounts of His-tagged p15^PAF^ were then incubated with PCNA-GST fusion protein in binding buffer (PBS containing 1% NP-40 and 2 mM DTT) for 3 h at 4 °C. The glutathione-sepharose beads, along with the PCNA-GST fusion protein and binding proteins, were collected by centrifugation (1 min, 16,000 *g*), washed extensively in binding buffer, and analyzed employing SDS-PAGE and western blotting using anti-p21 antibody.

## Results

### p15^PAF^ is expressed in proliferative tissues

**Figure 1:**
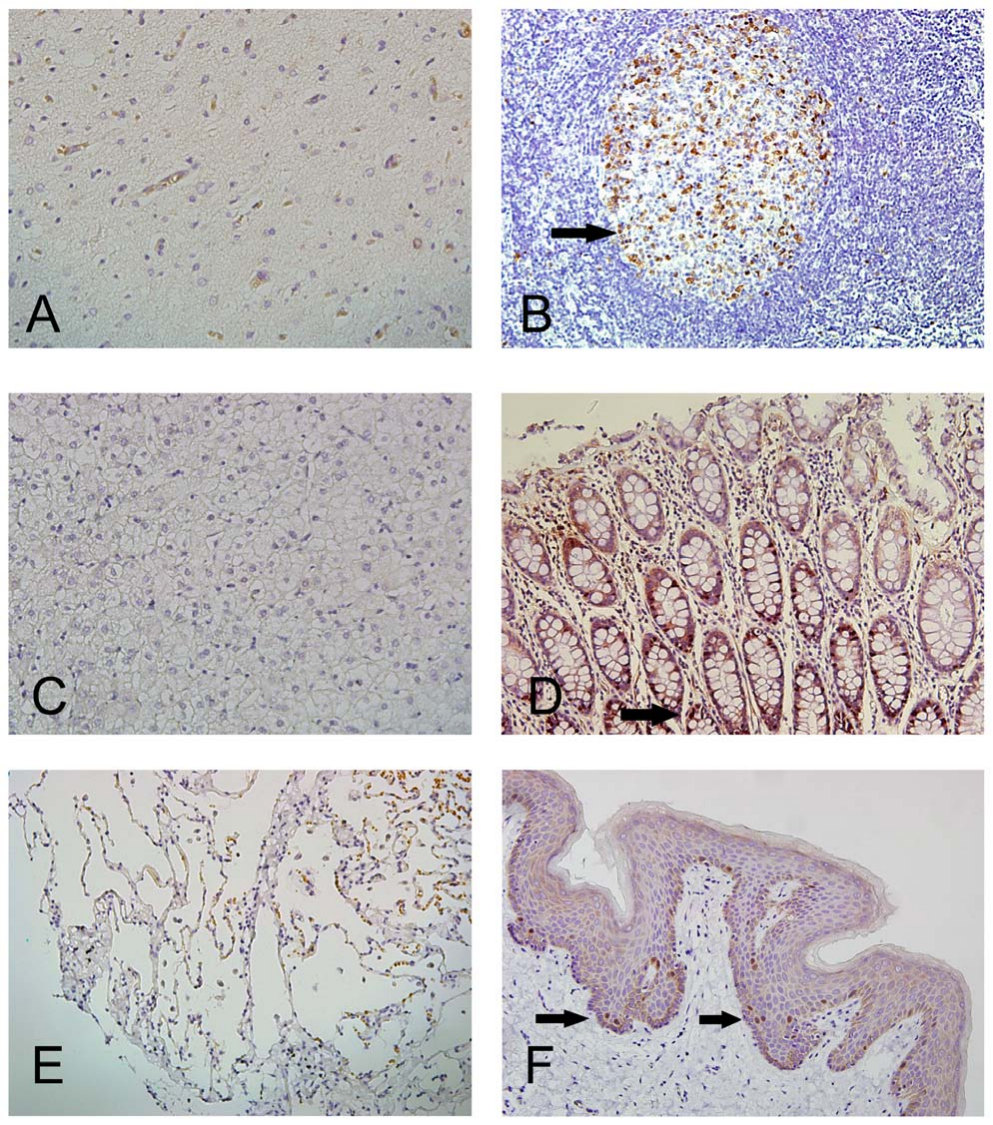
p15^PAF^ expression in normal adult tissues. Nuclear expression of p15^PAF^ was detected in the lymphocytes in the germinal center (arrow) of lymph node (B), the epithelial cells in the lower half of crypt (arrow) in the colonic mucosa (D), and the suprabasal keratinocytes (arrow) of epidermis (F), but not in the neurons and glial cells of brain (D), hepatocytes of liver (E), and (F) pneumocytes of lung.

To evaluate p15^PAF^ tissue distribution, we constructed a tissue array containing nontumorous adult tissues of major organs. Immunohistochemical staining revealed p15^PAF^ nuclear staining in the epidermis, lymph node, endometrium, and small and large intestines, but not in the brain, liver, heart, pancreas, lung, and myometrium (Fig. 1). In the lymph node, p15^PAF^ was specifically expressed in germinal centers. In the small and large intestines, p15^PAF^ was predominantly expressed in the lower half of the epithelial crypts, where proliferative cells reside. In the epidermis, p15^PAF^ was expressed in the suprabasal cells only, which are the major proliferative epidermal cells. This pattern of tissue distribution indicated that p15^PAF^ expression is restricted to proliferative cells.

### p15^PAF^ is an S-phase protein

**Figure 2:**
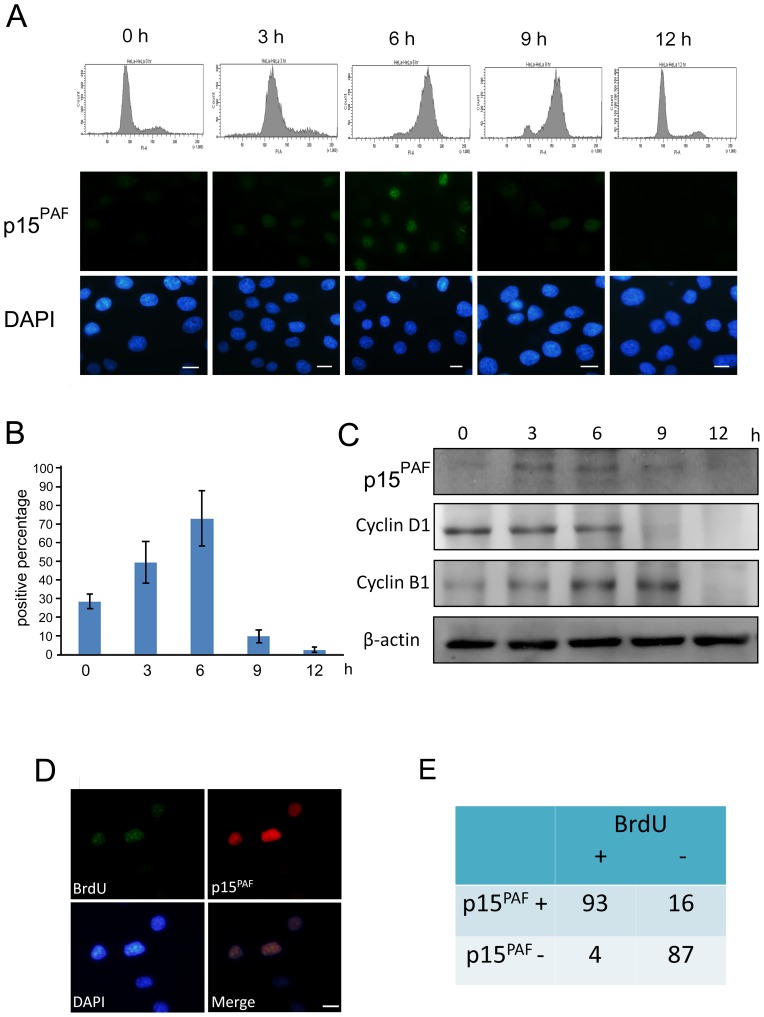
p15^PAF^ is expressed in S phase. (A) HeLa cells were synchronized at G1/S boundary by thymidine/aphidicholin double block. Immunofluorescence staining of p15^PAF^ was performed at the indicated time point after release. Flow cytometry was also performed to determine the cell cycle distribution. Expression of p15^PAF^ peaked at 6 h (mid-to-late S phase) after release. (Scale bar = 30 µm). (B) Quantification of the percentage of p15^PAF^-positive cells at the indicated time point. (C) HeLa cells were synchronized at the G1/S boundary thymidine/aphidicholin double block and were collected for Western blotting following release into the cell cycle. Cyclin D1 and B1 were used as markers of S and G2 phase, respectively. (D) After pulse labeling with BrdU, HeLa cells were stained with anti- p15^PAF^ and anti-BrdU antibodies. The cells with positive BrdU staining were also positive for p15^PAF^. (Scale bar = 30 µm). (E) Quantification of the result of immunofluorescence staining. 90% of the cells showed concordant staining of p15^PAF^ and BrdU.

To investigate the regulation of p15^PAF^ expression during the cell cycle, we synchronized HeLa cells at the G1/S boundary using the thymidine/aphidicolin double block method. We used immunofluorescence to determine the percentage of p15^PAF^-positive cells. As shown in Figs. 2A and 2B, p15^PAF^ expression peaked 6 h after release from aphidicolin, and rapidly declined 9 h after release, indicating the predominant expression of p15^PAF^ during the S phase. Western blotting also showed high p15^PAF^ expression 3 h and 6 h after release from aphidicolin, and downregulated p15^PAF^ expression 9 h after release (Fig. 2C). To verify this result, we pulse-labeled HeLa cells using 5-bromo-2′deoxy-uridine (BrdU). Immunofluorescence showed that nearly all cells with BrdU staining were also positive for p15^PAF^ and vice versa (90% concordant rate; Figs. 2D and 2E). These results indicated that p15^PAF^ is predominantly expressed during the S phase.

### p15^PAF^ is essential for cell cycle progression and DNA synthesis

**Figure 3:**
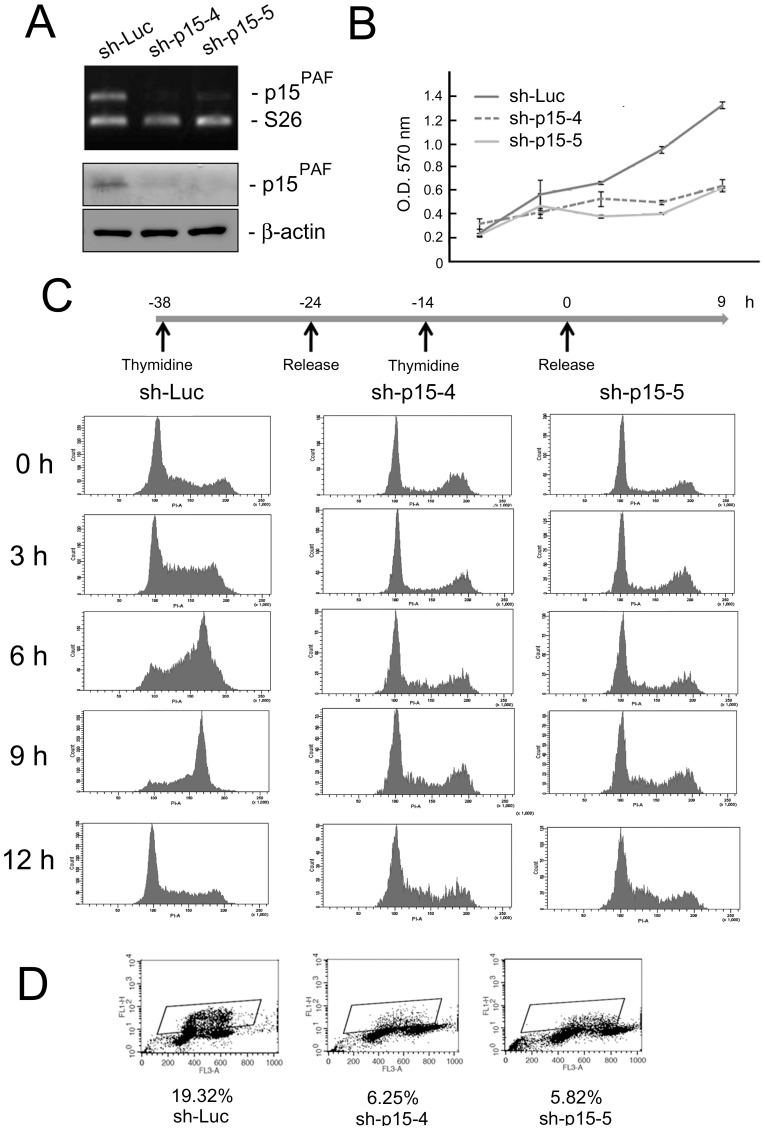
p15^PAF^ is essential for cell cycle progression and DNA synthesis. (A) p15^PAF^ mRNA (upper panel) and protein (lower panel) levels were significantly reduced in HeLa cells stably transduced with lentiviruses sh-p15-4 and -5. Sh-Luciferase (sh-Luc) served as the negative control. (B) The MTT proliferation assay revealed a growth inhibitory effect of knockdown of p15^PAF^. (C) Knockdown of p15^PAF^ suppressed cell cycle progression. After release from double thymidine block, the control cells progressed normally into S and G2 phases but the cells with knockdown of p15^PAF^ were arrested at G1 phase. (D). Knockdown of p15^PAF^ inhibited the incorporation of BrdU into DNA in S phase.

We obtained 5 different lentiviral constructs carrying p15^PAF^ shRNA from the National RNAi Core Facility (Academia Sinica, Taipei, Taiwan), and used them to transduce HeLa cells. Cells transduced with shRNAs #4 and #5 displayed marked reductions in p15^PAF^ mRNA and protein expression (Fig. 3A). We therefore used them in subsequent analyses. Using the MTT assay, we showed that stable knockdown of p15^PAF^ by shRNAs #4 and #5 led to the inhibition of HeLa cell proliferation (Fig. 3B). To evaluate the effects of p15^PAF^ knockdown on cell cycle progression, we synchronized HeLa cells at the G1/S boundary using a double thymidine block. As shown in Fig. 3C, the control cells progressed normally through the S phase to the G2 phase, and approximately one-third of cells completed the cell cycle and returned to the G1 phase 9 h after release from thymidine. In contrast, cells with attenuated p15^PAF^ expression remained at the G1/S boundary for up to 12 h after release. The major event that occurs during the S phase is DNA synthesis; therefore, we investigated the effects of p15^PAF^ knockdown on DNA synthesis. Our results from the BrdU incorporation assay showed that p15^PAF^ knockdown markedly inhibited DNA synthesis in asynchronous HeLa cells (Fig. 3D).

### p15^PAF^ knockdown upregulates p21 expression and increases its chromatin binding

**Figure 4:**
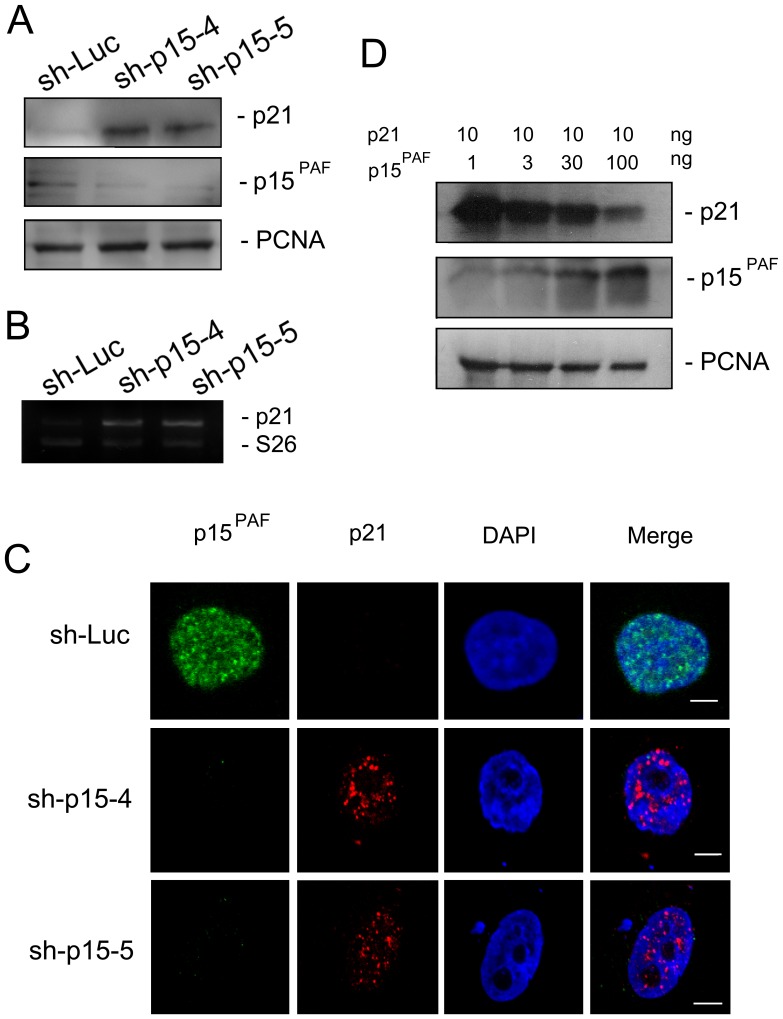
Knockdown of p15^PAF^ induces accumulation and chromatin binding of p21. (A) Western blotting showed the expression of p21 was enhanced by knockdown of p15^PAF^. (B) Expression of p21 mRNA was also enhanced by knockdown of p15^PAF^. (C) Immunofluorescence staining showed knockdown of p15^PAF^ enhanced chromatin binding of p21. (Scale bar = 5 µm). (D) In vitro GST-tagged protein-protein interaction assay also showed p15^PAF^ inhibited the binding of p21 to GST-PCNA fusion protein.

The cyclin-dependent kinase inhibitor p21^WAF1/CIP1^ (p21) plays important roles in the regulation of cell cycle progression. To elucidate the mechanism by which p15^PAF^ knockdown arrests the cell cycle, we evaluated p21 expression using western blotting. In unsynchronized HeLa cells, p15^PAF^ knockdown induced marked upregulation of the p21 protein (Fig. 4A) and mRNA expression (Fig. 4B). These results indicated that regulation of p21 expression is mainly at the transcriptional level. p21 is also a PCNA-interacting protein [11] and inhibits the ability of PCNA to activate DNA polymerase δ, the principal replicative DNA polymerase, which inhibits DNA replication [11]. We speculated that p15^PAF^ might compete with p21 for binding to PCNA, and that p15^PAF^ attenuation leads to excess interaction between p21 and PCNA. Therefore, we assayed the chromatin-bound fraction of p21 and identified that p15^PAF^ attenuation resulted in p21 accumulation in chromatin (Fig. 4C). An in vitro GST-tagged protein-protein interaction assay further showed that p15^PAF^ inhibited the binding of p21 to GST-PCNA fusion protein in a dose-dependent manner (Fig. 4D).

### p15^PAF^ expression is negatively regulated by the Rb/E2F pathway

**Figure 5:**
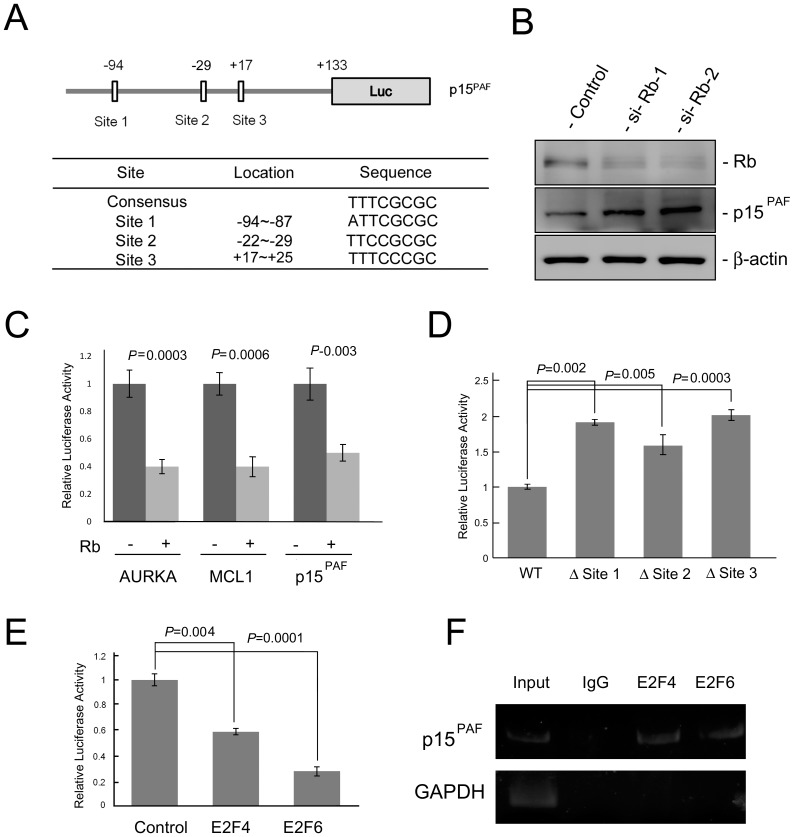
p15^PAF^ is a target of Rb/E2F pathway. (A) Three putative E2F-binding motifs are present in proximal promoter region of the p15^PAF^ gene. The promoter sequence containing these three putative E2F-binding motifs was cloned upstream to the luciferase (Luc) gene in the pGL3-Basic vector. (B) Knockdown of Rb by siRNA enhanced expression of p15^PAF^ in MCF7 cells. (C) Overexpression of Rb repressed the activity of p15^PAF^, AURKA, and MCL1 promoters. AURKA and MCL1 are known targets of the Rb/E2F pathway and served as positive controls. (D) Mutations of all three putative E2F-binding motifs derepressed the promoter activity of p15^PAF^. (E) E2F4 and E2F6 suppressed the promoter activity of p15^PAF^. (F) ChIP assay showed binding of E2F4 and E2F6 onto the p15^PAF^ promoter.

The Rb/E2F pathway is the major regulatory mechanism for genes that are required for S-phase entry, such as DNA polymerase subunits, cyclin A, and cyclin E [12]. Therefore, we hypothesized that p15^PAF^ is a direct transcriptional target of the Rb/E2F pathway. We analyzed the p15^PAF^ promoter sequence using the TFSEARCH website, identifying 3 putative E2F binding motifs located at −94 to −87, −29 to −22, and 17 to 25 bp of the transcriptional start site (Fig. 5A). HeLa cells have a defective Rb function caused by the binding of human papilloma virus protein E7 to Rb [13]; therefore, we used the breast cancer cell line MCF7 to evaluate Rb/E2F pathway-mediated p15^PAF^ regulation. As shown in Fig. 5B, following Rb attenuation by siRNA, p15^PAF^ expression was upregulated. This observation indicated that Rb is a negative regulator of p15^PAF^ expression. To validate the biological functionality of the 3 putative E2F motifs, we cloned the promoter sequence containing the 3 putative E2F-binding sites into a position upstream of the luciferase reporter gene in the plasmid pGL3-basic. Results from the luciferase assay showed the promoter activity was repressed by Rb (Fig. 5C). Rb also inhibited the promoters of 2 known Rb/E2F targets AURKA and MCL1 [14], [15]. To determine which E2F motifs are functional, we mutated each site using site-directed mutagenesis. When analyzing the effects of each mutation, we found that mutations in all 3 sites resulted in increased luciferase activity (Fig. 5D), indicating that all 3 sites are functional and that the Rb/E2F pathway exerts inhibitory effects on the p15^PAF^ promoter. Rb interacts with several E2F family members. Of these, E2F1–3 are transcriptional activators, whereas E2F4–8 are transcriptional repressors [16]. Cotransfection of E2F4 or E2F6 with the p15^PAF^ promoter reporter resulted in repressed promoter activity (Fig. 5E). We also performed chromatin immunoprecipitation (ChIP) to confirm the binding of E2F4 and E2F6 with the p15^PAF^ promoter in vivo (Fig. 5F). Overall, these results support the hypothesis that p15^PAF^ is a target of the Rb/E2F pathway.

## Discussion

p15^PAF^ is overexpressed in many types of solid tumor [2]–[6]. Previous studies have identified several functions for p15^PAF^, including DNA repair, cell proliferation, and tumor invasion [5]–[7], [17]. Using immunohistochemical staining, we found that p15^PAF^ is specifically expressed in proliferative cells in normal adult organs. Besides, p15^PAF^ is a PCNA-binding protein, so we propose that it is a component of cell proliferation machinery. In this study, we found that p15^PAF^ is expressed predominantly in the S phase, and is essential for S-phase progression and DNA synthesis.

The hypothesis that p15^PAF^ is involved in cell proliferation is not totally new. Consistent with our results, Mizutani et al. showed that p15^PAF^ knockdown markedly inhibited the growth of anaplastic thyroid cancer cells [18]. However, the distribution of p15^PAF^ during the cell cycle and its functional role in cell cycle progression remain controversial. Emanuele et al. concluded that p15^PAF^ expression peaks in the G2/M phase of the cell cycle and declines rapidly at the mitotic exit [19]. In contrast, we observed the predominant expression of p15^PAF^ during the S phase. We consider the discrepancies to be caused by differences in the interpretation of the results. In the figure 1C of their report, p15^PAF^ expression remained at high levels from 0 h to 7 h after release from the double thymidine block, and declined 8 h after release, coinciding with the appearance of the G2/M marker phospho-serine 10 on histone 3. This expression pattern is more consistent with that of an S-phase protein than that of a G2/M protein. In our study, we used immunofluorescence and western blotting to evaluate the cell cycle distribution. We identified that p15^PAF^ expression is highly concordant with BrdU incorporation, indicating that p15^PAF^ is predominantly expressed during the S phase. Besides, in the study by Emanuele et al., p15^PAF^ attenuation led to a decreased cell number during the S phase [19]. This observation was consistent with our result that p15^PAF^ knockdown inhibited S-phase progression. Therefore, although we cannot exclude p15^PAF^ function during other cell cycle phases, our results indicate that its main function is in promoting S-phase progression.

In this study, we observed that p15^PAF^ knockdown upregulated p21 expression. p21 protein has 2 major mechanisms for cell cycle arrest. It binds to and inhibits the activity of cyclin-dependent kinases CDK1, 2, and 4, to induce G1 arrest and block entry into the S phase [20]. The p21 is also a PCNA-interacting protein and inhibits the activation of DNA polymerase δ by PCNA [11].We observed that p15^PAF^ knockdown upregulated p21 expression and increased the levels of p21 in the chromatin-bound fraction, indicating that p15^PAF^ is able to compete with p21 for binding to PCNA. Consistent with our results, Yu et al. observed that p21 overexpression reduced p15^PAF^ binding to PCNA, and that p15^PAF^ upregulation inhibited the binding of p21 to PCNA [21]. Therefore, equilibrium between p21 and p15^PAF^ expression might be an important mechanism in the determination of S-phase entry.

Our study results from luciferase assays, ChIP experiments, and transient transfection assays clearly show that E2F4 and E2F6 repressed transcription from the p15^PAF^ promoter by binding to the proximal promoter region. These observations supported our hypothesis that p15^PAF^ expression varies during cell cycle progression. Many E2F target genes, such as p15^PAF^, have complex promoter structures that include 2 or more E2F consensus sites [22], [23]. In some E2F target genes, the activating and repressing E2Fs bind to the same or different E2F-binding motifs to regulate gene expression [24]. In p15^PAF^, we observed that all 3 E2F-binding motifs repressed p15^PAF^ expression. However, whether each site has a distinct function in the control of p15^PAF^ expression remains unknown.

In summary, our results indicate that p15^PAF^ is an S-phase protein tightly regulated by the Rb/E2F complex, and that loss of Rb/E2F-mediated inhibition during the G1/S transition leads to upregulated p15^PAF^ expression. p15^PAF^ then competes with p21 for binding to PCNA. Therefore, the presence of p15^PAF^ is essential for DNA synthesis and S-phase progression.
